# Hospitalized Patient as Source of *Aspergillus fumigatus*, 2015

**DOI:** 10.3201/eid2408.171865

**Published:** 2018-08

**Authors:** Baptiste Lemaire, Anne-Cécile Normand, Jean-Marie Forel, Nadim Cassir, Renaud Piarroux, Stéphane Ranque

**Affiliations:** Assistance Publique–Hôpitaux de Marseille, Marseille, France (B. Lemaire, J.-M. Forel, N. Cassir, S. Ranque);; Assistance Publique–Hôpitaux de Paris, Paris, France (A.-C. Normand, R. Piarroux);; Aix-Marseille Université, Marseille (J.-M. Forel, S. Ranque);; Institut Hospitalo-Universitaire Méditerranée Infection, Marseille (N. Cassir, S. Ranque);; Sorbonne Université, Paris (R. Piarroux)

**Keywords:** Aspergillus fumigatus, intensive care units, airborne contamination, France, fungi, hospital infections, aspergillosis

## Abstract

Hospital-acquired aspergillosis is usually associated with environmental contamination. In 2015, continuous monitoring of airborne fungi and multilocus variable-number tandem-repeat analysis identified the source of *Aspergillus fumigatus* as the airway of a patient. Therefore, patients colonized with *Aspergillus* spp. should be treated in airborne infection isolation rooms.

Pulmonary aspergillosis is acquired by inhalation of airborne spores in the environment. Hospital-acquired aspergillosis is usually associated with airborne fungal contamination of the hospital environment, especially after building construction events. A previous report described *Aspergillus fumigatus* transmission from an intensive care unit (ICU) patient with a sporulating liver-transplant surgical site infection to 2 other patients (secondary pulmonary aspergillosis) (*1*). We describe a patient whose respiratory tract was colonized by *A. fumigatus*; we found no reports of hospital environment contamination by this organism. Continuous monitoring of indoor airborne fungal contamination with electrostatic dustfall collectors (EDCs) (*2**–**5*) demonstrated that the airway of a patient was the point source of airborne *A. fumigatus* contamination in the ICU.

## The Case

On July 29, 2015, a 61-year-old man was hospitalized in the ICU at the University Hospital of Marseille (Marseille, France) with acute respiratory distress syndrome as a complication of a lung abscess. The patient had a history of smoking tobacco (1 pack/d for 25 y), a nonrepaired herniated lumbar disk, and recent periodontitis. At the time of admission, the microbiological workup results, including bacteriological and mycological culture (of blood, urine, bronchial aspirate, and superficial swab sample assessment for possible fungal colonization) and HIV serology, were negative. Chest radiographs showed bilateral interstitial pneumonitis and abscessation of the right lower lung lobe. Bronchial fibroscopy showed inflammatory mucosa. The following were initiated: mechanical ventilation with a system equipped with antimicrobial filters, extra corporeal membrane oxygenation, and antibacterial therapy (imipenem, ciprofloxacin, and vancomycin). Anemia, hypoalbuminemia, and a clotting disorder subsequently developed. On day 20, a tracheotomy was performed. The patient’s airways were moisturized by water vapor from a humidification system without a filter that allowed airflow between room air and the patient’s airways. No filamentous fungus was detected after culture of 9 bronchoalveolar lavage and 11 bronchial aspirate samples. The following bacteria were isolated: *Porphyromonas endodontalis* (day 5), *Klebsiella pneumoniae* (day 10), and *Pseudomonas aeruginosa* showing intermediate resistance to imipenem (day 36). Antibacterial therapy was modified to intravenous meropenem plus intrabronchial colistin and then adjusted to tazocillin and tobramycin. On days 52 and 55, *Candida albicans* was isolated from 2 blood cultures and a double-J ureteric stent sample. Caspofungin treatment was started and continued for 2 weeks. No respiratory samples were analyzed during the following months. 

On day 114, the patient experienced hemoptysis. A diagnosis of pulmonary aspergillosis was based on the following criteria: positive culture, *A. fumigatus* detection in a sputum sample culture, positive galactomannan antigen (index = 4.27 in respiratory samples), and bilateral nodules visible on chest computed tomography image. During the following 47 days, 5 bronchial aspirate samples grew *A. fumigatus* on culture, and 4 of 6 samples were positive for galactomannan antigen. Seven serum samples were negative for galactomannan antigen.

Because severe liver dysfunction contraindicated the use of triazoles, 3 mg/kg/d of anti-*Aspergillus* liposomal amphotericin B was initiated on day 116. On day 134, a chest computed tomography image showed bilateral pulmonary nodules, some of which displayed a halo sign. The liposomal amphotericin B dosage was then increased to 5 mg/kg/d. Of note, during days 118–160, the patient’s airways were directly connected to his environment during several attempts to wean him off mechanical ventilation. The patient continued receiving liposomal amphotericin B without substantial clinical improvement. Bacteremia with *Staphylococcus aureus* and *Bacteroides thetaiotaomicron* developed and was treated with vancomycin and imipenem. The patient died on day 169.

**Figure 1:**
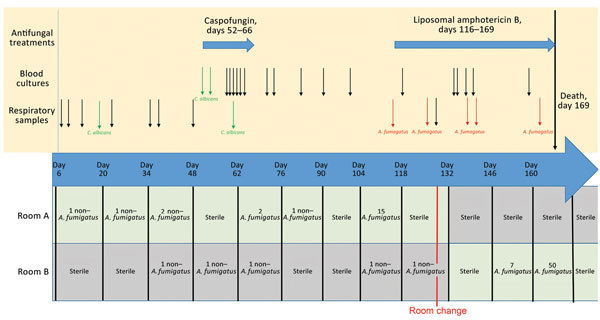
Timeline summarizing the antifungal treatments, patient blood and respiratory sample mycology culture results, and filamentous fungi culture results of the electrostatic dustfall collectors used for continuous monitoring of airborne fungal contamination in the intensive care unit rooms where *Aspergillus fumigatus* was found during hospital stay of colonized patient, France, 2015. *C. albicans*, *Candida albicans*.

EDCs were used to continuously monitor fungal airborne contamination in the ICU for 244 weeks; each EDC was exposed for 14 days. EDCs have been used in various indoor environments to detect and quantify cultivable microorganisms and a wide range of airborne analytes, including endotoxins, allergens, β-glucans, and microbial DNA (*2**–**4*). EDC-based measurements provide an accurate qualitative and quantitative profile of the cultivable airborne fungal communities present during a given sampling period (*5*). The patient’s first room (room A) remained free of airborne *A. fumigatus* contamination from the day the patient entered the room to day 62 (Figure 1). *A. fumigatus* airborne contamination was detected during the 2-week periods of days 62–76 (2 CFU) and days 104–118 (15 CFU). On day 128, the patient was transferred from room A to room B. Room A was disinfected and thereafter remained free of airborne fungal contamination. Fifteen days after the room change, airborne *A. fumigatus* contamination was detected in room B.

**Figure 2:**
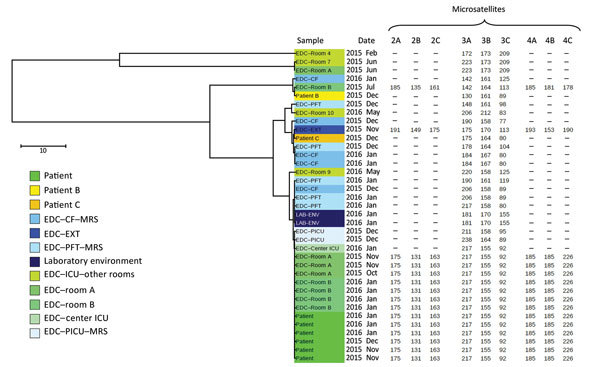
Multilocus variable-number tandem-repeat analysis genotyping results for *Aspergillus fumigatus* found during hospital stay of colonized patient, France, 2015. The distance tree was plotted by using iTOL version 4.2 (https://itol.embl.de/), taking into account the 3 microsatellite markers that were obtained from each *A. fumigatus* isolate sampled from the various study sites. The length-polymorphisms of 9 microsatellite markers were obtained for the 14 isolates sampled at the ICU during the patient’s stay and 1 control. ICU room locations are detailed in the Technical Appendix. CF, cystic fibrosis center; EDC, electrostatic dustfall collector; EXT, a school in northeastern France; ICU, intensive care unit; MRS, Marseille; PFT, pulmonary function testing center; PICU, pediatric intensive care unit. Scale bar indicates nucleotide substitutions per site.

All 39 *A. fumigatus* isolates collected in the ICU since the beginning of 2015 were genotyped by using a variable number of tandem repeats assay as previously described (*6*). Genotyping revealed that the *A. fumigatus* strain isolated from the sputum sample on day 116 and from the air sample collected from room A by EDC during days 62–76 was the same (Figure 2). Because no respiratory samples were collected during days 62–116, concomitant *A. fumigatus* colonization of the patient was not documented. Subsequently, the same strain was isolated from the EDC in room A during days 104–118 and several times from the EDCs placed in room B after the patient was transferred (Figures 1, 2). In summary, the 6 *A. fumigatus* strains isolated from the patient’s airway and the 8 air samples from the patient’s room shared the same multilocus genotype. In contrast, 2 *A. fumigatus* strains isolated during routine monitoring of the ICU for airborne fungal contamination before the patient was admitted to the ICU (the first was isolated 7 weeks earlier in room A; the second, 3 weeks earlier in room B) had 0/9 and 1/9 alleles, respectively, in common with the strain from the patient. Airborne *A. fumigatus* contamination was not detectable in room B after the patient died, although no disinfection had been performed.

## Conclusions 

Continuous monitoring of airborne fungal contamination by using EDCs combined with multilocus variable-number tandem-repeat analysis genotyping traced the source of *A. fumigatus* contamination in this case. By using these 2 complementary approaches, we showed that the *A. fumigatus* strain had not been detected in room A before the patient’s admittance. Although the patient might have been carrying this *A. fumigatus* strain before ICU admittance, it is more likely that it was hospital acquired because *A. fumigatus* was not detected in the first 9 respiratory samples tested. No respiratory samples were tested during the 6 weeks after the first positive EDC culture; thus, the time when the *A. fumigatus* colonization of the patient’s airway became detectable could not be ascertained. Nevertheless, the same strain further colonized the patient’s airway before he was transferred to room B, where the level of airborne contamination became highly significant; up to 50 CFU were cultured from 1 EDC in room B. Furthermore, in the absence of concomitant construction work or other known risk factors for hospital fungal contamination, we found increased contamination levels just before the patient died. Taken together, these observations support the hypothesis that the patient acquired the infection during his stay in room A and his airway then became the point source of airborne *Aspergillus* contamination of room B. The genotyping results further support the hypothesis that the patient’s airway was the point source of airborne *Aspergillus* contamination of room B. Overall, these findings strongly argue that patients colonized with *Aspergillus* spp. should undergo treatment exclusively in airborne-infection isolation rooms, especially when patients at risk for aspergillosis are hospitalized in the same unit.

Technical Appendix Map of the intensive care unit where patient with *Aspergillus fumigatus* stayed, France, 2015.
