# Supplementary material for: Hospitalized Patient as Source of Aspergillus fumigatus, 2015
Source: Emerg Infect Dis. 2018 Aug;24(8):1524–7. doi: 10.3201/eid2408.171865 (PMC6056103; doi:10.3201/eid2408.171865)
Supplement: Technical Appendix — Map of the intensive care unit where patient with Aspergillus fumigatus stayed, France, 2015. [file 17-1865-Techapp-s1.pdf]

Article DOI: https://doi.org/10.3201/eid2408.171865

Hospitalized Patient as Source of *Aspergillus fumigatus*, 2015

Technical Appendix

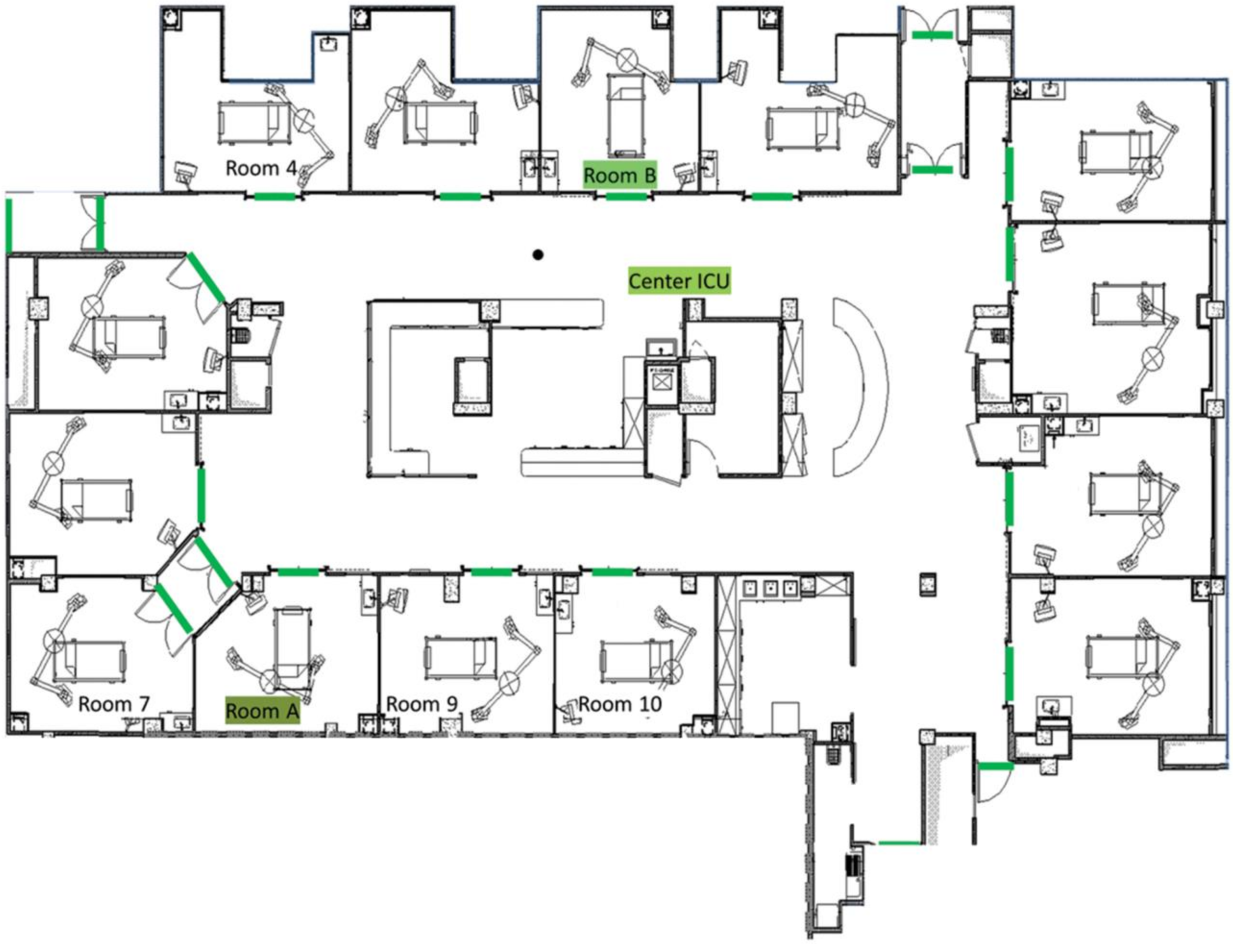

Technical Appendix Figure. Map of the intensive care unit, indicating the locations of rooms A and B where the patient stayed and the other rooms where *Aspergillus fumigatus* isolates were sampled and genotyped.
